# Evaluation of the Health-Promoting Properties of Selected Fruits

**DOI:** 10.3390/molecules26144202

**Published:** 2021-07-10

**Authors:** José A. Figueira, Priscilla Porto-Figueira, Cristina Berenguer, Jorge A. M. Pereira, José S. Câmara

**Affiliations:** 1 CQM—Centro de Química da Madeira, Campus da Penteada, Universidade da Madeira, 9020-105 Funchal, Portugal; jose.figueira@staff.uma.pt (J.A.F.); priscilla.figueira@staff.uma.pt (P.P.-F.); cristina.berenguer@staff.uma.pt (C.B.); jorge.pereira@staff.uma.pt (J.A.M.P.); 2 Departamento de Química, Faculdade de Ciências Exatas e da Engenharia, Campus da Penteada, Universidade da Madeira, 9020-105 Funchal, Portugal

**Keywords:** fruits, nutraceutical, antioxidant, antidiabetic, antihypertensive

## Abstract

In this study, the health-promoting benefits of different fruits grown in Madeira Island, namely lemon (*Citrus limon* var. *eureka*), tangerine (*Citrus reticulata* var. *setubalense*), pitanga (*Eugenia uniflora* var. *red*), tomato (*Solanum lycopersicum* var. *gordal*) and uva-da-serra, an endemic blueberry (*Vaccinium padifolium* Sm.), were investigated. The phenolic composition (total phenolics and total flavonoids content) and antioxidant capacity (assessed through ABTS and DPPH assays) were measured revealing a high phenolic potential for all fruits, except tomato, while uva-da-serra is particularly rich in flavonoids. In relation to the antioxidant capacity, the highest values were obtained for pitanga and uva-da-serra extracts. The bioactive potential was also assessed through the ability of the extracts to inhibit digestive enzymes linked to diabetes (α-amylase, α- and β-glucosidases) and hypertension (angiotensin-converting enzyme, ACE). The results obtained point to a very high bioactive potential with the selected samples exhibiting very important ACE anti-enzymatic capacities. A statistical analysis of the obtained data reveals a very strong correlation between ABTS and TPC, and a strong contribution of the fruit polyphenols for enzyme inhibition, and thus, presenting high antihypertensive and antidiabetic capacities. Overall, the results obtained clearly show a high bioactive potential of the selected fruits that should be further studied, in terms of specific phenolic composition. Moreover, these results strongly support the valorisation of pitanga seeds usually discarded as a waste, and uva-da-serra, an endemic and wild bush, as potential bioresources of bioactive compounds with impact in human diet.

## 1. Introduction

It is widely-known that the consumption of fruits and vegetable elicit health protection against different diseases. According to Aune et al. [1], between 5.6 and 7.8 million premature deaths occurring worldwide in 2013, were attributable to a low fruit, and vegetable intake (lower than 500 and 800 g/day), respectively. In this context, Cheung et al. [2] pointed that most of these mortality risks, mainly those related to cardiovascular disease, chronic diseases, and cancer, could be reduced by regular and varied consumption of fruit and vegetables. These protective effects are largely attributed to secondary metabolites including polyphenols, glucosinolates, carotenoids, terpenoids, alkaloids, saponins, vitamins, among others, present in fruits and vegetables [3] exhibiting antioxidant, antiatherogenic, anti-inflammatory, antimicrobial, and cardioprotective effects [4]. These bioactive compounds are mostly produced by plants to cope with different challenges particularly those related to adverse environmental conditions (hydric stress, high temperatures and humidity levels). In this sense, Madeira Island has very challenging climate conditions, with very hot and humid conditions all over the year, high thermic variations and pronounced slopes which correlate with the high bioactive potential and complex volatile compositions exhibited by fruits growing in Madeira Island in comparison with same varieties from other geographical regions. Previously, we have shown that tomato (*Solanum lycopersicum* L.) from Gordal variety grown in Madeira Island presented higher tocopherols content when compared with other varieties commonly consumed like Campari, Roma and cherry [5]. In addition, recently, Detopoulou et al. [6] underlined the relevance of tocopherols, as vitamin E, on the proper functioning of the immune system, acting as an antioxidant in the context of the importance of phytochemicals of the Mediterranean diet against COVID-19 effects. Similarly, polyphenols have been suggested as a therapeutic adjuvant in the treatment of COVID-19 patients [7]. These classes of secondary metabolites with high nutraceutical value are widely found in nature, including in citrus fruits. Among them, flavonoids have been previously associated with a positive effect in the treatment of different diseases, including arthritis, diabetes mellitus, cancer and neurodegenerative disorders, as well as liver, kidney and heart diseases [8]. Overall, this protection, elicited by metabolites, presenting in citrus fruits contributes to strengthening their general awareness of functional foods [9]. For instance, lemon is very rich in a large variety of secondary metabolites, mainly monoterpenes, which are used in nutraceutical and food industries [10]. Tangerines are another widely consumed citrus fruit that contains a similar bioactive content profile to lemon, namely in what concerns monoterpenes [9]. According to Figueira et al. [9,10], both lemons and tangerines, grown in Madeira Island, were shown to have a very complex volatile composition with some of identified VOCs being responsible for health benefits. Tutunchi et al. [11] and Alberca et al. [12] reported the potential effects of naringenin, a flavanone with antiviral and anti-inflammatory activities, as a promising treatment strategy against COVID-19.

*Vaccinium padifolium* Sm. is an endemic blueberry tree from Madeira Island, locally known as uveira-da-serra. Interest and consumption of its berries have increased in recent years due to its very high nutritional value [13], related to the high content of phenolic compounds [14,15]. 

*Eugenia uniflora* L. fruits, popularly known as “Suriname cherry” or “pitanga” is an exotic fruit native from Brazil [16], but widely available in Madeira Island. Pitanga is appreciated by consumers for its softness, aromatic and bittersweet flavour, and presents a low lipid and caloric content and high amounts of phenolic compounds [17,18], carotenoids and anthocyanins [19]. Several ethnomedical uses of *E. uniflora* have been documented, especially those related to leaves and oils extracts [20], which present in vitro antiproliferative potential [21]. The leaves and fruits extracts have also shown stimulant, febrifuge, aromatic and antidiarrheal characteristics [22], and pitanga juices, anti-inflammatory properties [23]. However, Pitanga seeds are usually discarded. Although, Oliveira et al. [24] reported on the promising antioxidant potential requiring further studies to explore its different fields of application. 

In this context, this study aimed to evaluate the health-promoting proprieties of fruits of regular consumption grown in Madeira Island, including the total phenolic and flavonoid contents (TPC and TFC, respectively), antioxidant capacity (ABTS and DPPH assays) and its ability to inhibit digestive enzymes linked to diabetes (α-amylase, α- and β-glucosidase) and hypertension (angiotensin-converting enzyme). The fruits were selected according to its high bioactive potential, previously reported, namely tomato (gordal variety) [5,25], lemon (eureka variety) [10], tangerine (setubalense variety) [9], uva-da-serra [15], and pitanga. Liquid-liquid-based ultrasound-assisted extraction (LLUSAE), an efficient extraction procedure for vegetable matrices, previously optimized in our lab [25], was used to obtain the extracts. 

## 2. Materials and Methods

### 2.1. Chemicals and Materials

Angiotensin-converting enzyme (ACE, from human, 95%), hydrochloric acid (HCl, ACS reagent, 37%), trisodium citrate dihydrate (C_6_H_5_Na_3_O_7_·2H_2_O, 99%), fluorescein, 2,2′-azobis(2-methylpropionamidine) dihydrochloride (AAPH), sodium carbonate (Na_2_CO_3_), 3,5-Dinitrosalicylic acid (DNS) color reagent, aluminium chloride (AlCl_3_), sodium nitrite (NaNO2 ACS reagent, 97.0%), α- and β-glucosidase, α- and β-pNPG, and α-amylase were acquired from Sigma-Aldrich (Buchs, Switzerland). Potassium phosphate dibasic trihydrate (K_2_HPO_4_·3H_2_O) was acquired from Merck (Buchs, Switzerland), ethanol (EtOH, absolute PA, 99.5%), potassium dihydrogen phosphate (KH_2_PO_4_, 99.5%), sodium chloride (NaCl, 99.8%), *N*-[3-(2-furyl)acryloyl]-Phe-Gly-Gly (FAPGG) and sodium hydroxide (NaOH) were acquired from Panreac (Barcelona, Spain). Folin-Ciocalteu solution, 6-Hydroxy-2,5,7,8-tetramethylchroman-2-carboxylic acid (trolox, 98%), and 2,2-Diphenyl-1-picrylhydrazyl (DPPH) were acquired from Fluka (Munich, Germany). Acetonitrile (ACN) and methanol (MeOH) (both HPLC grade, 99.99%) from Thermo Fisher Scientific (Loughborough, UK), and PSA/C18/Mg_2_SO_4_ (25/25/150 mg, DisQuE) was purchased from Waters (Milford, MA, USA). The ultrapure water used on the assays was obtain using an Ultrapure water purification system (Milli-Q^®^ Direct 8 at 18 MW cm, 23 °C, Millipore Corporation, Burlington, MA, USA).

### 2.2. Samples

**Figure 1:**
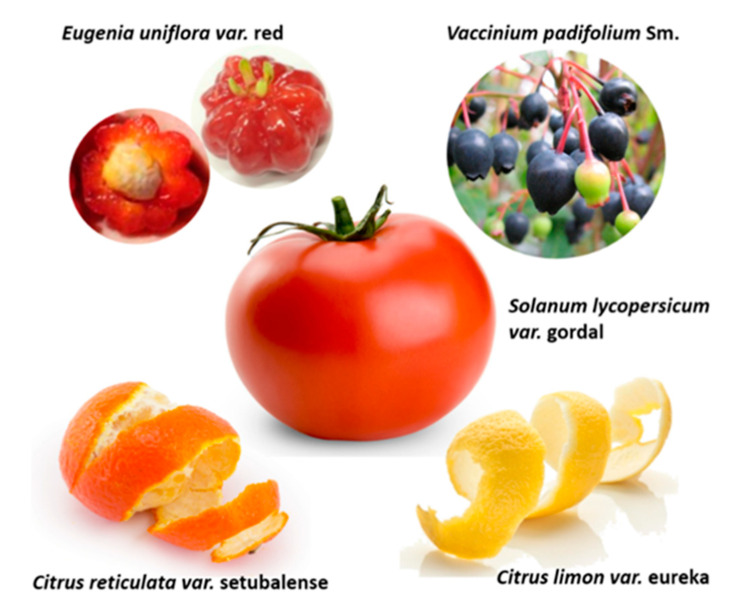
Fruits under study: Pitanga (*Eugenia uniflora* variety red) seeds and pulp, uva-da-serra (*Vaccinium padifolium* Sm.), tomato (*Solanum lycopersicum* variety gordal), lemon (*Citrus limon* variety *eureka*) peels and juice and tangerine (*Citrus reticulata* variety setubalense) peels and juice.

Lemon samples (eureka variety), tangerine (setubalense variety) and tomato (gordal variety) were selected randomly from a local market (Madeira Island), as purchased for consumption (Figure 1). Red pitanga and uva-da-serra samples were randomly harvested directly from trees in local productions in Madeira Island (Figure 1). After selection, five hundred grams of the peels and juice from lemon and tangerines, the seeds and pulp from pitanga and the whole tomato and uva-da-serra fruits were collected and immediately stored under N_2_ (g) atmosphere at −80 °C. Then, with exception of the citrus juices, all samples were lyophilized (Christ Alpha 1–2 LD plus freeze dryer, Osterode am Harz, Germany), grounded to powder (IKA A11 basic analytical mill, Staufen, Germany) and immediately stored under nitrogen at −80 °C, in several aliquots. All aliquots were used only once to prevent sample degradation. 

### 2.3. Extraction

Sample extraction was performed according to the procedure previously optimized for vegetable matrices by Figueira et al. [25]. Briefly, 50 mg of the lyophilized samples for each fruit were diluted with 9 mL of ACN:MeOH (4:1, *v/v*), sonicated (BRANSON 2510E-DTH, 100 W, 40 kHz, Danbury, CT, USA) for 30 min at 25 °C and centrifuged during 5 min (5000× *g*, Espresso Personal microcentrifuge, Thermo Scientific, Waltham, MA, USA). Finally, the supernatant was collected, homogenized in the vortex, centrifuged again (5 min, 5000× *g*), cleaned up with 180 mg of primary secondary amine (PSA)/C18/Mg_2_SO_4_ (1:1:6; *w*, *w*, *w*)/mL of sample extract (the sorbent was homogenized in the supernatant by vortexing and centrifuged 5 min, 5000× *g*) and filtrated (0.2 μm) before analysis.

### 2.4. Evaluation of Bioactive Potential

#### 2.4.1. Total Phenolic Content (TPC) and Total Flavonoid Content (TFC)

The TPC was determined using a modified Folin-Ciocalteu procedure. Briefly, fruit extracts were diluted in water up to 1 mL final volume, added 100 µL of Folin-Ciocalteu solution, 400 µL of Na_2_CO_3_ (20%), and 500 µL of water. After 1h, the electron transfer from phenolic compounds is measured by UV-Vis at λ = 765 nm. TFC assay was performed using the aluminium chloride colorimetric method. Briefly, fruits extracts were diluted in methanol (70%) up to 1 mL final volume, added 60 µL of NaNO_2_ (5%) and rest 5 min in the dark. Then, added 60 µL AlCl_3_ (10%), rest another 5 min before the addition of 400 µL of NaOH (1 M), a 2-min rest and finally 480 µL of methanol (70%). The acid-stable complexes formed by the AlCl_3_ with flavones and flavonols were measured at λ = 510 nm.

#### 2.4.2. Total Antioxidant Capacity (TAC)

DPPH assay was performed according to Figueiraet al. [25]. Briefly, 500 µL methanol was added to the fruit extracts, followed by 1 mL of free-radical 2,2-diphenyl-1-picrylhydrazyl (DPPH) and stored 10 min in the dark, before UV-Vis analysis at λ = 515 nm (Lambda 25, Perkin Elmer, Belgium) to measure the free-radical reduction. ABTS assay was adapted from the procedure reported by Thaipong et al. [26]. Briefly, a stock solution of 2,29-azinobis-(3-ethylbenzothiazoline-6-sulfonic acid) radical cation (ABTS, 7.3 mM and potassium persulfate 2.59 mM) was prepared in ethanol and rest in the dark for 16h, at room temperature, before use. Ethanol was added to the fruits extracts up to a 100 µL final volume and added 1.9 mL of 100× diluted ABTS solution (in ethanol). After 2 h storage in the dark, the reduction of the radical cation was measured at 734 nm. 

#### 2.4.3. Antihypertensive Capacity

The antihypertensive capacity was assessed using the ACE-inhibition activity assay reported by Holmquist, et al. [27] with minor changes. Briefly, 50 µL of FAPGG (2 mM) were diluted in 450 µL of Tris-HCl buffer (50 mM, with 300 mM of NaCl and HCl 0.1 M at pH 8.3). After homogenization by vortex (1 min), 400 µL of water were added, then 50 µL of the sample, followed by homogenization before adding 50 µL of ACE (125 mU diluted from a stock solution of 25 U in the phosphate-potassium buffer—KH_2_PO_4_ 9.3 mM and K_2_HPO_4_·3H_2_O 0.7 M; with 300 mM NaCl at pH 8.3), and incubate 3 min at 37 °C. Finally, the absorbance was measured every 2 min for 20 min at λ = 328 nm.

#### 2.4.4. Antidiabetic Capacity

The study of the antidiabetic capacity was estimated through the inhibition of the digestive enzymes α- and β-glucosidases and α-amylase. For α- and β-glucosidases assay, 50 µL of the respective enzyme (1 U/mL) were added to 25 µL of the sample extract and incubated 10 min at 37 °C. A total of 100 µL of the substrate α-pNPG (5 mM) or β-pNPG (5 mM), respectively, were added and incubated 30 min at 37 °C.

The reaction was terminated by adding 180 µL of Na_2_CO_3_ (0.1 M) and the absorbance measured at λ = 405 nm. α-Amylase inhibition was evaluated by adding 400 µL of the substrate starch (1%) to 200 µL of the sample extract followed by 3 min incubation at 37 °C, and addition of 200 µL of the enzyme (13 U/mL) followed by a new 3 min of incubation at 37 °C. It was adicionad200 µL of DNS color reagent (DNS 96 mM, potassium sodium tartrate 5.31 M in NaOH 2 M), finally, the mixture was incubated at 95 °C for 10 min in a dry bath (Block heating system Grant QBD1, Frilabo, Portugal), the reaction stopped with the addition of 900 µL of cold water, and absorbance measured at λ = 540 nm. 

### 2.5. Statistical Analysis

The statistical analysis was performed using the MetaboAnalyst 4.0 web-based tool [28] (https://www.metaboanalyst.ca/ (accessed on 9 July 2021)). The raw data obtained in the bioactive assays (TPC, TFC, ABTS, DPPH, α-amylase, α-glucose, β-glucose, and ACE-inhibition) was pre-processed by normalization (to sample median, data transformation by Log_10_ normalization and data scaling by autoscaling). Additionally, a one-way Analysis of Variance (ANOVA, *p* < 0.05) was carried out, followed by post-hoc analyses Tukey’s Honestly Significant Difference (Tukey’s HSD, *p* ≤ 0.05), used for mean comparisons among dates. The correlations between variables were examined by Pearson’s correlation (*p* < 0.05).

## 3. Results and Discussion

**Figure 2:**
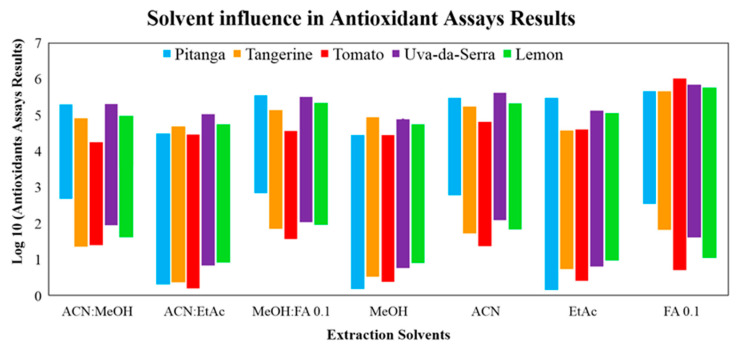
The influence of the extraction conditions on the antioxidants assays performance was assessed by measuring the TAC, TPC and TFC for each fruit extract (pitanga, tangerine, tomato, uva-da-serra and lemon) obtained using each of the seven solvent extraction conditions: methanol—MeOH, acetonitrile—ACN, ethyl acetate—EtAc, formic acid 0.1%—FA, ACN:MeOH (4:1, *v/v*), ACN:EtAc (1:1, *v/v*) and MeOH:FA (19:1, *v/v*). Log_10_ was applied to results obtained normalize the data obtained and allow a better comparison of the best extraction condition.

In previous works, a high concentration in different bioactive compounds, particularly phenolic compounds, has been observed in the fruits analysed in this work [5,9,10,15,25]. This observation led us to investigate the bioactive potential of the juice and peels of lemon and tangerine, pulp and seeds of pitanga, and the whole fruit of tomato and uva-da-serra. The bioactivity was assessed by measuring the phenolic content (total phenolic content, TPC, and total flavonoid content, TFC), the antioxidant capacity (total antioxidant assays, TAC, DPPH and ABTS), as well the inhibition of key enzymes associated with antihypertensive and antidiabetic effects. To allow a systematic comparison between the fruit extracts, seven extraction conditions were assayed to find the most suitable to all samples. Accordingly, TPC and TFC were determined for 35 different conditions (five sample extracts vs. seven extraction conditions). The data obtained (Supplementary Materials; Table S1) were normalized as log_10_ of the antioxidant assays values determined to allow the identification of the best extraction conditions. As shown in Figure 2, the solvent mixtures ACN:MeOH (4:1, *v*/*v*), MeOH:FA (19:1, *v/v*) and ACN retrieve the best results. ACN:MeOH (4:1, *v/v*) was the selected condition as it was previously used with success in the extraction of hydrophilic and lipophilic bioactive compounds (carotenoids or tocopherols) [5,25].

### 3.1. Phenolic Content and Antioxidant Capacity of the Selected Fruits Extracts

**Figure 3:**
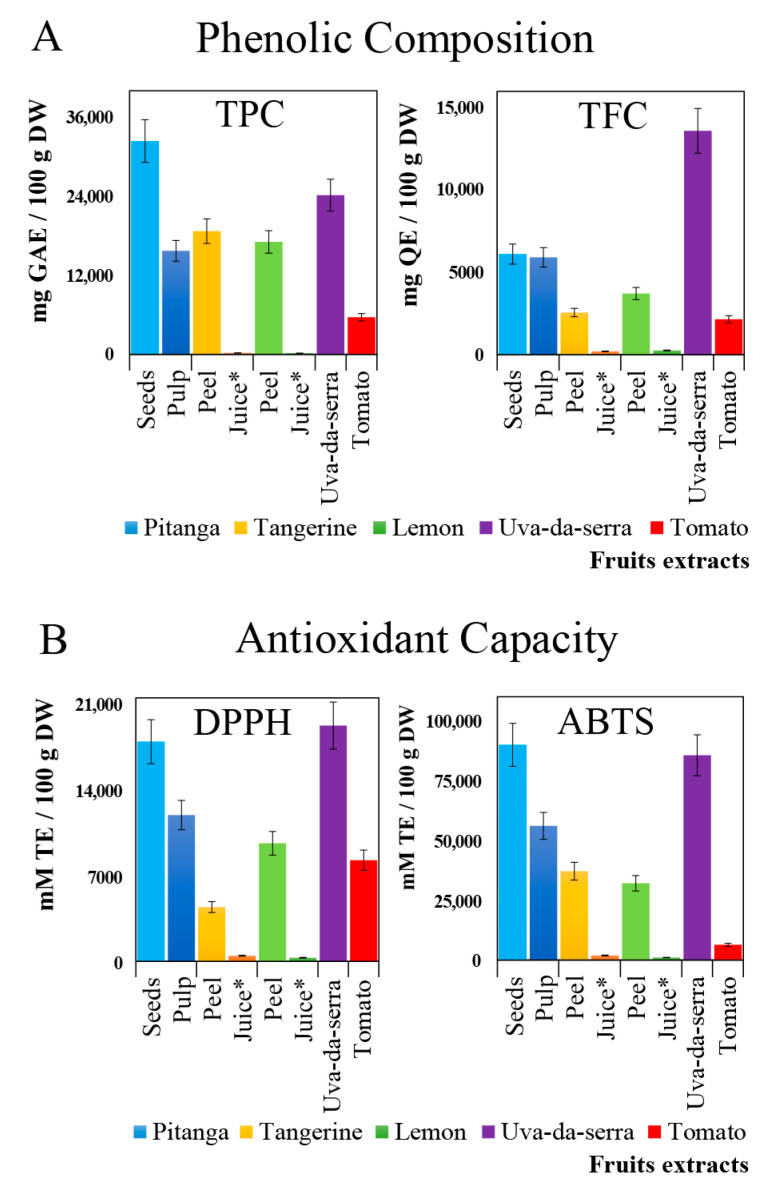
Evaluation of the phenolic composition (**A**) and antioxidant capacity (**B**) of the selected fruit extracts, pitanga (seeds and pulp), tangerine (peel and juice), lemon (peel and juice), uva-da-serra (whole fruits) and tomato (whole fruits). The phenolic composition was evaluated through the assessment of the total phenolic composition (TPC) and total flavonoid content (TFC), while the total antioxidant capacity (TAC) was evaluated using DPPH and ABTS assays; * by 100 mL of fresh juice instead of dry weight (DW). Legend: ABTS—2,29-azinobis-(3-ethylbenzothiazoline-6-sulfonic acid) radical cation assay, ACE—angiotensin-converting enzyme, DPPH—2,2-diphenyl-1-picrylhydrazyl free radical assay, GAE—gallic acid equivalents, QE—quercetin equivalents, TFC—total flavonoid content, TE—Trolox equivalents, TPC—total phenolic content.

The TPC and TFC were determined to get a snapshot of the phenolic amount of the selected fruits. As shown in Figure 3A, pitanga seeds and uva-da-serra berries exhibit the highest phenolic content, while peels from lemon and tangerines and the pulp from pitanga also contain very interesting TPCs, being much lower for tomato and vestigial in tangerine and lemon juices. Although most of these individual results are in agreement with the previous literature results for uva-da-serra [14], tangerine [29] and lemon [30] and tomato [31], however, pitanga seeds present a TPC that is being, to the best of our knowledge, reported for the first time.

Very relevant is the high flavonoid composition of uva-da-serra which is almost three times higher than the levels found in pitanga seeds, the second extract with the highest TFC value (Figure 3A, right panel). Again, this result is corroborated by our previous results [15] which revealed that 8 of the top 10 free low molecular polyphenols identified in uva-da-serra are flavonoids. To understand the impact of the TPC and TFC values found in the selected fruits, a literature survey was performed to compare the results obtained with the ones previously reported in other studies involving the same or similar fruits (Supplementary Materials, Table S2). A systematic comparison among all data collected is not totally feasible due to the variations in the experimental conditions used in the different reports, namely the fruits extracts preparation and extraction technique. Despite that fact, the potential of the fruits studied in this work is very relevant and promising as they present the richest phenolics content and antioxidant capacities. To unveil putative correlations between the high phenolic content and antioxidant capacity, the antioxidant capacity of the 8 extracts analysed was assessed through DPPH and ABTS assays. As Figure 3B shows, the antioxidant capacity of pitanga (seeds and pulp) and uva-da-serra replicate the trends observed for the phenolic content, being these extracts that present the highest values for the DPPH and ABTS assays. The data obtained is supported by previous reports for the different fruits studied, namely tomato [31], uva-da-serra [14], pitanga [22] and lemon [32,33]. Detailed data is listed in Table S2.

Overall, the TAC results point very clearly to the promising antioxidant proprieties of the endemic blueberry uva-da-serra and pitanga. Moreover, the obtained results show that the seeds of pitanga, which are not edible and generally discarded as waste, can be explored as a bioresource of natural antioxidants and nutraceuticals unveiling important applications in the food industry as a bioresource of natural antioxidant compounds.

### 3.2. Enzymatic Inhibition Capacity

**Figure 4:**
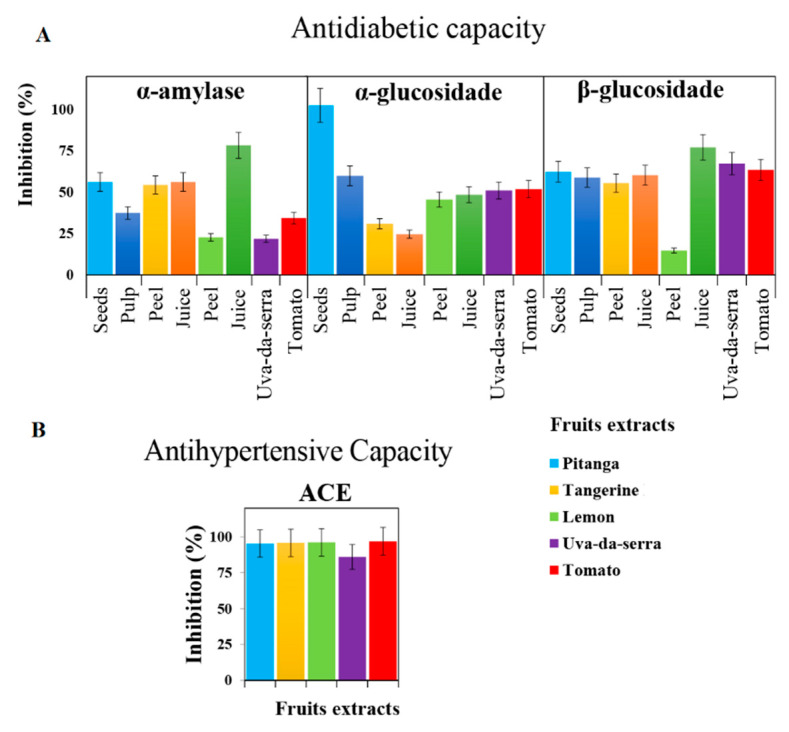
In vitro inhibitory activities (of the selected fruits samples towards (**A**) antidiabetic- (α-amylase—83 µL extract/1 U, α- and β-glucosidase—500 µL extract/1 U) and (**B**) antihypertensive- (ACE—8 µL extract/1 U)) model enzymes. For legend simplification, fruits sections were organized by colors: pitanga seeds and pulp in light and dark blue, respectively, tangerine peel and juice in yellow, and light orange, respectively, lemon peel and juice in light and dark green, respectively. Legend: ACE—Angiotensin-converting enzyme.

To obtain further evidence of the bioactive potential of the fruits analysed beyond their high antioxidant properties, enzyme inhibition assays were performed using selected enzymes to verify putative antidiabetic and antihypertensive effects. The antidiabetic potential was estimated through the inhibition of the digestive enzymes α- and β-glucosidases and α-amylase. The α-glucosidase and α-amylase are the main enzymes that mediate the metabolism of dietary carbohydrates [34]. Also, α- and β-glucosidase are responsible for the conversion of glycosidic bond into oligosaccharide and finally into monosaccharide [35]. Accordingly, the inhibition of these enzymes will delay glucose absorption, preventing post-meal peaks of glucose in blood that eventually trigger diabetes development [35]. The results obtained (Figure 4) reveal a high antidiabetic potential, mostly above 50% inhibition of α- and β-glucosidases and α-amylase. Pitanga seeds extracts, however, are particularly effective against α-glucosidase, reaching a total inhibitory effect. 

The antihypertensive capacity of the selected extracts was assessed through the ACE-inhibition activity assay. As can be observed in Figure 4, except for uva-da-serra, which presents an inhibitory effect of around 90%, the remaining fruit extracts achieved almost full ACE-inhibition. Overall, these results agree with previous observations taking into account that fruits rich in flavonoids, as the ones studied, exert important inhibitory effects on ACE [36]. 

### 3.3. Statistical Analysis

To unveil a possible correlation between the phenolic composition, antioxidant capacity and antihypertensive and antidiabetic potential for the selected fruits extracts, correlation coefficients (r) between the TPC and TFC assays, TAC, and enzyme inhibition of ACE, α -amylase and α- and β-glucosidases were performed (Table 1).

According to the correlation matrix obtained, DPPH presents a strong inverse correlation to ABTS and TFC, and a moderate inverse correlation to TPC. In fact, DPPH assay is very sensitive to the nature of the antioxidants present in the extracts analysed and may manifest positive or negative correlations with those [37].

**Figure 5:**
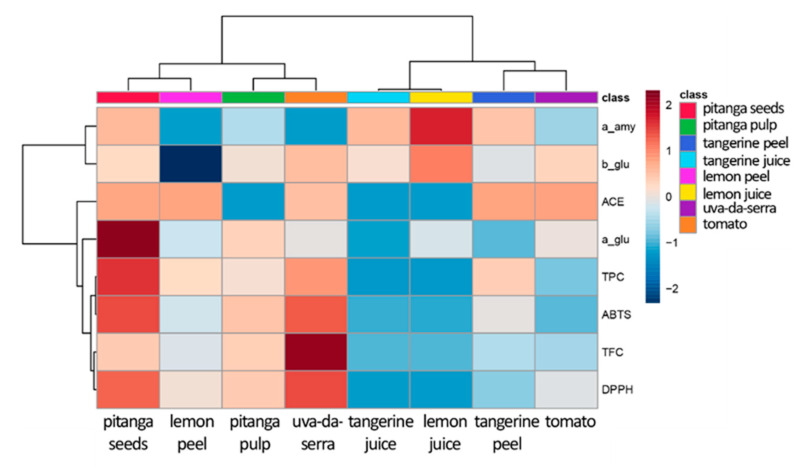
Correlation heatmap to evaluate the putative contribution of the phenolics and flavonoids present in the samples extracts to the TAC and key enzymes inhibition. The different assays were performed as described in Materials and Methods. Legend: a_amy—α-amylase, a_glu—α-glucosidase, b_glu—β-glucosidase, ABTS—2,29-azinobis-(3-ethylbenzothiazoline-6-sulfonic acid) radical cation assay, ACE—angiotensin-converting enzyme, DPPH—2,2-diphenyl-1-picrylhydrazyl free radical assay, TAC—total antioxidant capacity, TFC—total flavonoid content, TPC—total phenolic content.

In turn, there is a strong correlation between ABTS and TPC. Regarding the enzymatic inhibition assays, there is a strong correlation between themselves, which suggests that the nutraceuticals presented in the samples may have common anti-enzymatic effects. To further understand the translation of these correlations in the selected fruit extracts, correlations heatmaps were produced for each sample (Figure S1). The results obtained are evidence of important variations in the correlation between phenolic composition, antioxidant capacity and enzyme inhibition that were summarized in an overall correlation heatmap (Figure 5). Accordingly, it is easily observed that the contribution of the phenolics of each fruit extract to the antioxidant capacity is very different among fruits (uva-da-serra vs tomato, for instance), as well as among fruit sections (pitanga seeds vs pulp or tangerine and lemon peels vs. the respective juices). In relation to the enzyme inhibition assays, Figure 1 reveals a strong contribution of the phenolics present in pitanga seeds to the inhibition of the selected enzymes. Such behaviour is different from what can be observed for tangerine, lemon or uva-da-serra, in which the respective phenolics seems to be more effective against one enzyme than another. As an example of such correlation, such phenolic composition is not very effective in the inhibition of α-amylase or ACE despite the higher phenolic composition of the uva-da-serra extracts, which correlate with a high antioxidant capacity. In contrast, an overall positive correlation is particularly evident for pitanga seed extracts that present the highest phenolic content (Figure 3A), highest antioxidant capacity (Figure 3B) and highest enzyme inhibition (Figure 5). Similar correlation patterns were previously reported in citrus fruits by Alu’datt, et al. [38]. 

## 4. Conclusions

In this work, the health-promoting benefits of different fruits grown in Madeira Island—lemon (*Citrus limon* var. eureka), tangerine (*Citrus reticulata* var. setubalense), pitanga (*Eugenia uniflora* var. red), tomato (*Solanum lycopersicum* var. gordal) and uva-da-serra, (*Vaccinium padifolium* Sm.), were investigated to evaluate its bioactive potential based on TPC, TFC, antioxidant capacities, antihypertensive and anti-diabetic properties. Overall, the analysis of seeds and pulp of pitanga, and peels and juices tangerine and lemon, uva-da-serra and tomato, reveal a high bioactive potential that justifies further and deeper studies uncovering the specific nutraceutical composition, namely the phenolic content of the fruits studied. This is particularly relevant in the context of the production of new functional foods with antihypertensive and antidiabetic capacities. In this context, pitanga seeds, which are inedible, at least in their raw presentation, and thus, discarded as waste, have a great potential and should be further explored. In turn, uva-da-serra, the berry of the wild bush *Vaccinium padifolium* is fairly unknown and so there is also a great potential in its use in the human diet, taking into consideration the high bioactive potential determined in this study.

## Figures and Tables

**Table 1:** Pearson correlation coefficients between phenolic composition, antioxidant capacity and selected enzymes inhibition for the fruit extracts analysed in this work.

DPPH	TFC	TPC	ABTS	β-glu	α-glu	α-amy	ACE
1.00	−0.80	−0.65	−0.83	0.22	0.13	0.02	−0.11
	1.00	0.27	0.57	−0.21	−0.27	−0.33	−0.12
		1.00	0.92	0.48	0.59	0.74	0.77
			1.00	0.35	0.29	0.47	0.51
				1.00	0.75	0.81	0.72
					1.00	0.92	0.96
						1.00	0.94
							1.00

Legend: α-amy—α-amylase, α-glu—α-glucosidase, β-glu—β-glucosidase, ABTS—2,29-azinobis-(3-ethylbenzothiazoline-6-sulfonic acid) radical cation assay, ACE—angiotensin-converting enzyme, DPPH—2,2-diphenyl-1-picrylhydrazyl free radical assay, TAC—total antioxidant capacity, TFC—total flavonoid content, TPC—total phenolic content.

## Data Availability

The data presented in this study are available upon reasonable request from the corresponding author.

## Supplementary Materials

The following are available online, Table S1: The influence of the extraction conditions on the antioxidants assays for each fruit extract, Table S2: Literature survey of the bioactivity of the selected fruits, Figure S1: Correlation heatmaps between the bioactive assays for each fruit extract.

## Sample Availability

Samples of the compounds are not available from the authors.

## References

[1] Aune D., Giovannucci E., Boffetta P., Fadnes L.T., Keum N., Norat T., Greenwood D.C., Riboli E., Vatten L.J., Tonstad S. (2017). Fruit and vegetable intake and the risk of cardiovascular disease, total cancer and all-cause mortality—a systematic review and dose-response meta-analysis of prospective studies. Int. J. Epidemiol..

[2] Cheung J.T.H., Lok J., Gietel-Basten S., Koh K. (2021). The Food Environments of Fruit and Vegetable Consumption in East and Southeast Asia: A Systematic Review. Nutrients.

[3] Mannucci C., Casciaro M., Sorbara E., Calapai F., Di Salvo E., Pioggia G., Navarra M., Calapai G., Gangemi S. (2021). Nutraceuticals against Oxidative Stress in Autoimmune Disorders. Antioxidants.

[4] Câmara J.S., Albuquerque B.R., Aguiar J., Corrêa R.C.G., Gonçalves J.L., Granato D., Pereira J.A.M., Barros L., Ferreira I.C.F.R. (2020). Food Bioactive Compounds and Emerging Techniques for Their Extraction: Polyphenols as a Case Study. Foods.

[5] Figueira J.A., Pereira J., Câmara J.S. (2017). Quantification of δ-, γ- and α-Tocopherol in Tomatoes Using an Improved Liquid-Dispersive Solid-Phase Extraction Combined with Ultrahigh Pressure Liquid Chromatography. Food Anal. Methods.

[6] Detopoulou P., Demopoulos C., Antonopoulou S. (2021). Micronutrients, Phytochemicals and Mediterranean Diet: A Potential Protective Role against COVID-19 through Modulation of PAF Actions and Metabolism. Nutrients.

[7] El-Missiry M.A., Fekri A., Kesar L.A., Othman A.I. (2021). Polyphenols are potential nutritional adjuvants for targeting COVID-19. Phytother. Res..

[8] Ahmed O.M., AbouZid S.F., Ahmed N.A., Zaky M.Y., Liu H. (2021). An Up-to-Date Review on Citrus Flavonoids: Chemistry and Benefits in Health and Diseases. Curr. Pharm. Des..

[9] Figueira J.A., Porto-Figueira P., Pereira J.A., Câmara J.S. (2020). Tangerines Cultivated on Madeira Island—A High Throughput Natural Source of Bioactive Compounds. Foods.

[10] Figueira J.A., Porto-Figueira P., Pereira J., Câmara J.S. (2020). A comprehensive methodology based on NTME/GC-MS data and chemometric tools for lemons discrimination according to geographical origin. Microchem. J..

[11] TuTunchi H., Naeini F., Ostadrahimi A., Hosseinzadeh-Attar M.J. (2020). Naringenin, a flavanone with antiviral and anti-inflammatory effects: A promising treatment strategy against COVID-19. Phytother. Res..

[12] Alberca R.W., Teixeira F.M.E., Beserra D.R., De Oliveira E.A., Andrade M.M.D.S., Pietrobon A.J., Sato M.N. (2020). Perspective: The Potential Effects of Naringenin in COVID-19. Front. Immunol..

[13] Carvalho M.J., Gouveia C.S., Vieira A.C., Pereira A.C., de Carvalho M.A.A.P., Marques J.C. (2017). Nutritional and Phytochemical Composition of Vaccinium padifoliumSm Wild Berries and Radical Scavenging Activity. J. Food Sci..

[14] Spínola V., Pinto J., Castilho P.C. (2018). Hypoglycemic, anti-glycation and antioxidant in vitro properties of two Vaccinium species from Macaronesia: A relation to their phenolic composition. J. Funct. Foods.

[15] Figueira J.A., Porto-Figueira P., Pereira J.A.M., Câmara J.S. Fingerprint of the free low molecular weight phenolics compo-sition and bioactivity of Vaccinium padifolium Sm. fruits. Food Res. Int..

[16] Bicas J.L., Molina G., Dionísio A.P., Barros F.F.C., Wagner R., Maróstica M.R., Pastore G.M. (2011). Volatile constituents of exotic fruits from Brazil. Food Res. International..

[17] Rodrigues A.C., Zola F.G., Ávila Oliveira B.D., Sacramento N.T.B., Da Silva E.R., Bertoldi M.C., Taylor J.G., Pinto U.M. (2016). Quorum Quenching and Microbial Control through Phenolic Extract of Eugenia Uniflora Fruits. J. Food Sci..

[18] Siebert D.A., De Mello F., Alberton M.D., Vitali L., Micke G.A. (2019). Determination of acetylcholinesterase and α-glucosidase inhibition by electrophoretically-mediated microanalysis and phenolic profile by HPLC-ESI-MS/MS of fruit juices from Brazilian Myrtaceae Plinia cauliflora (Mart.) Kausel and Eugenia uniflora L.. Nat. Prod. Res..

[19] Biazotto K.R., Mesquita L.M.D.S., Neves B.V., Braga A., Tangerina M.M.P., Vilegas W., Mercadante A.Z., De Rosso V.V. (2019). Brazilian Biodiversity Fruits: Discovering Bioactive Compounds from Underexplored Sources. J. Agric. Food Chem..

[20] Sobeh M., El-Raey M., Rezq S., Abdelfattah M.A., Petruk G., Osman S., El-Shazly A.M., El-Beshbishy H.A., Mahmoud M., Wink M. (2019). Chemical profiling of secondary metabolites of Eugenia uniflora and their antioxidant, anti-inflammatory, pain killing and anti-diabetic activities: A comprehensive approach. J. Ethnopharmacol..

[21] Aranha E.S.P., de Azevedo S.G., dos Reis G.G., Lima E.S., Machado M.B., de Vasconcellos M.C. (2019). Essential oils from Eugenia spp.: In vitro antiproliferative potential with inhibitory action of metalloproteinases. Ind. Crop. Prod..

[22] Santos D.N., de Souza L.L., de Oliveira C.A.F., da Silva E.R., de Oliveira A.L. (2015). Arginase inhibition, antibacterial and antioxidant activities of Pitanga seed (Eugenia uniflora L.) extracts from sustainable technologies of high pressure extraction. Food Biosci..

[23] Soares D.J., Walker J., Pignitter M., Walker J.M., Imboeck J.M., Ehrnhoefer-Ressler M.M., Brasil I.M., Somoza V. (2014). Pitanga (Eugenia uniflora L.) fruit juice and two major constituents thereof exhibit anti-inflammatory properties in human gingival and oral gum epithelial cells. Food Funct..

[24] Oliveira A.L., Destandau E., Fougère L., Lafosse M. (2014). Isolation by pressurised fluid extraction (PFE) and identification using CPC and HPLC/ESI/MS of phenolic compounds from Brazilian cherry seeds (Eugenia uniflora L.). Food Chem..

[25] Figueira J.A., Pereira J.A., Porto-Figueira P., Câmara J.S. (2017). Ultrasound-assisted liquid-liquid extraction followed by ultrahigh pressure liquid chromatography for the quantification of major carotenoids in tomato. J. Food Compos. Anal..

[26] Thaipong K., Boonprakob U., Crosby K., Cisneros-Zevallos L., Byrne D.H. (2006). Comparison of ABTS, DPPH, FRAP, and ORAC assays for estimating antioxidant activity from guava fruit extracts. J. Food Compos. Anal..

[27] Holmquist B., Bünning P., Riordan J.F. (1979). A continuous spectrophotometric assay for angiotensin converting enzyme. Anal. Biochem..

[28] Chong J., Soufan O., Li C., Caraus I., Li S., Bourque G., Wishart D.S., Xia J. (2018). MetaboAnalyst 4.0: Towards more transparent and integrative metabolomics analysis. Nucleic Acids Res..

[29] Goldenberg L., Yaniv Y., Porat R., Carmi N. (2017). Mandarin fruit quality: A review. J. Sci. Food Agric..

[30] Lu S.-Y., Chu Y.-L., Sridhar K., Tsai P.-J. (2021). Effect of ultrasound, high-pressure processing, and enzymatic hydrolysis on car-bohydrate hydrolyzing enzymes and antioxidant activity of lemon (Citrus limon) flavedo. LWT.

[31] Aguiar J., Gonçalves J.L., Alves V.L., Câmara J.S. (2020). Chemical Fingerprint of Free Polyphenols and Antioxidant Activity in Dietary Fruits and Vegetables Using a Non-Targeted Approach Based on QuEChERS Ultrasound-Assisted Extraction Combined with UHPLC-PDA. Antioxidants.

[32] Raspo M.A., Vignola M.B., Andreatta A.E., Juliani H.R. (2020). Antioxidant and antimicrobial activities of citrus essential oils from Argentina and the United States. Food Biosci..

[33] Gironés-Vilaplana A., Moreno D.A., García-Viguera C. (2014). Phytochemistry and biological activity of Spanish Citrus fruits. Food Funct..

[34] Leyva-López N., Gutiérrez-Grijalva E.P., Vazquez-Olivo G., Heredia J.B. (2017). Essential Oils of Oregano: Biological Activity beyond Their Antimicrobial Properties. Molecules.

[35] Ilyas Z., Shah H.S., Al-Oweini R., Kortz U., Iqbal J. (2014). Antidiabetic potential of polyoxotungstates: In vitro and in vivo studies. Metallomics.

[36] Błaszczak W., Jeż M., Szwengiel A. (2020). Polyphenols and inhibitory effects of crude and purified extracts from tomato varieties on the formation of advanced glycation end products and the activity of angiotensin-converting and acetylcholinesterase enzymes. Food Chem..

[37] Rey F., Zacarías L., Rodrigo M.J. (2020). Carotenoids, Vitamin C, and Antioxidant Capacity in the Peel of Mandarin Fruit in Relation to the Susceptibility to Chilling Injury during Postharvest Cold Storage. Antioxidants.

[38] Alu’Datt M.H., Rababah T., Alhamad M.N., Al-Mahasneh M.A., Ereifej K., Al-Karaki G., Al-Duais M., Andrade J.E., Tranchant C.C., Kubow S. (2017). Profiles of free and bound phenolics extracted from Citrus fruits and their roles in biological systems: Content, and antioxidant, anti-diabetic and anti-hypertensive properties. Food Funct..

