# Supplementary material for: Evaluation of the Health-Promoting Properties of Selected Fruits
Source: Molecules. 2021 Jul 10;26(14):4202. doi: 10.3390/molecules26144202 (PMC8306905; doi:10.3390/molecules26144202)

Supplementary Material

Table S1. The influence of the extraction conditions on the antioxidants assays (TAC, TPC and TFC) for each fruit extract (pitanga, tangerine, tomato, uva-da-serra and lemon). Data results were normalized by Log_{10} application.

Antioxidant Assay	Extraction Solvent	Pitanga	Tangerine	Tomato	Uva-da-Serra	Lemon
**DPPH**	ACN:MeOH	2.1	0.51	0.78	1.2	0.85
	ACN:EtAc	0.29	0.35	0.19	0.81	0.90
	MeOH:FA 0.1	2.3	1.1	0.82	1.3	1.2
	MeOH	0.17	0.50	0.37	0.75	0.88
	ACN	2.2	0.96	0.82	1.32	1.0
	EtAc	0.13	0.72	0.40	0.79	0.96
	FA 0.1%	2.0	1.1	0.69	1.59	1.0
**TPC**	ACN:MeOH	5.5	5.2	4.6	5.4	5.2
	ACN:EtAc	4.5	4.7	4.5	4.7	4.7
	MeOH:FA 0.1	5.7	5.4	4.7	5.6	5.5
	MeOH	4.4	4.9	4.44	4.9	4.7
	ACN	5.6	5.3	4.7	5.7	5.5
	EtAc	4.5	4.6	4.6	4.7	4.7
	FA 0.1%	5.7	5.1	4.9	5.5	5.8
**TFC**	ACN:MeOH	4.7	3.8	3.3	5.1	4.3
	ACN:EtAc	3.9	4.0	4.2	5.0	4.1
	MeOH:FA 0.1	5.0	4.4	4.1	5.3	4.7
	MeOH	2.9	3.5	2.8	4.6	3.8
	ACN	5.0	4.9	4.8	5.4	4.9

	EtAc	5.5	4.5	4.3	5.1	5.0
	FA 0.1%	5.4	5.8	6.0	5.8	5.5
**ABTS**	ACN:MeOH	4.3	3.9	3.1	4.3	3.9
	ACN:EtAc	-	-	-	-	-
	MeOH:FA 0.1	4.3	4.2	3.7	4.3	4.3
	MeOH	-	-	-	-	-
	ACN	4.3	3.9	2.9	4.3	4.2
	EtAc	-	-	-	-	-
	FA 0.1%	4.2	3.8	-	-	-

Solvent extraction conditions: methanol - MeOH, acetonitrile – ACN, ethyl acetate - EtAc, formic acid 0.1 % - FA, ACN:MeOH (4:1, v/v), ACN:EtAc (1:1, v/v) and MeOH:FA (19:1, v/v).

Table S2. Literature survey of Total phenolic content (TPC), total flavonoid content (TFC), total antioxidant capacity (DPPH and ABTS assays) and inhibition of selected enzymes (α-amylase, α- and β-glucosidase, and angiotensin-converting enzyme (ACE), reported for pitanga, tangerine, lemon, tomato and uva-da-serra extracts.

**Extracts conditions**	**TPC**	**TFC**	**DPPH**	**ABTS**	**α-amylase[1]**	**α-glucosidase[1]**	**β-glucosidase[1]**	**ACE[1]**	**Ref**
**Pitanga**									
Seeds / pulp	32373 / 15707 mg GAE / 100 g DW	6112 / 5912 mg QE / 100 g DW	17845 / 11883 mM TE / 100 g DW	89907 / 56066 mM TE / 100 g DW	56 / 37	103 / 60	62 / 59	95	This work
oils from leaves			15350 - 40030 mg TE / 100 g						1
supercritical extract of seeds	0.003-7.3 mg GAE / 100 g		(IC_{50}) - 460.1 - 5024.6 mg / 100 g	532-3337 μM TE / 100 g					2
pressurized fluid extraction of seeds (dried- 12.7% H_2O)	420-1680 mg GAE / 100 g DW								3
pulp homogenized in ethanolic solution	663.8 mg CAE / 100 g		IC_{50} - 15.45 μg / mL			IC_{50} - Acarbose 413.6 μg / mL, extract 0.26 μL / mL			4
	517-908 mg CAE / 100 g FW		IC_{50} – 212 - 317 μg / mL			IC_{50} - Acarbose 413.6 μg / mL, extract 66-212 μg / mL			5
	226.88 mg CAE / 100 mL		85.9 % DPPH inhibition			74.2			6
juice	36.7 mg GAE / 100 mL					69.47 % inhibition with 5 mg / mL			7

pulp homogenized in methanolic solution	4253 mg CAE / 100 g		20668 mM TE / 100 g		IC_{50} - 5.7 mg / mL reaction solution	IC_{50} - 1.15 mg / mL reaction solution			8
leaves	19306 mg GAE / 100 g	2864 mg GAE / 100 g							9
**Tangerine**									
Peel / juice[2]	18692 / 216[2] mg GAE / 100 g DW	2559 / 202[2] mg QE / 100 g DW	4408 / 457[2] mM TE / 100 g DW	37126 / 1905[2] mM TE / 100 g DW	54 / 56	31 / 25	56 / 60	96	This work
oil								59.2	10
oil			750-2000 mg TE / 100 mL	1550-3000 mg TE / 100 mL					11
juices			24-68 % DPPH inhibition						12
juices	92 mg GAE / 100 mL	11 mg GAE / 100 mL	64.53 % DPPH inhibition	0.98 % ABTS inhibition		IC_{50} - 12 mg / mL			13
freeze dried			2.50 mM TE / 100g DW	6.47 mM TE / 100 g DW					14
**Lemon**									
Peel / juice[2]	17067 / 175[2] mg GAE / 100 g DW	3715 / 256[2] mg QE / 100 g DW	9592 / 294[2] mM TE / 100 g DW	32081 / 1033[2] mM TE / 100 g DW	23 / 78	46 / 48	15 / 77	96	This work
-	4750 mg / 100 g		94.4% DPPH inhibition		87.3				15
oil								24.8	10
lyophilized juice								18	16
oil			2000-2750 mg TE / 100 mL	600-1500 mg TE / 100 mL					11

oil	1951-3197 mg GAE / 100 g	229-414 mg QE / 100g	49.29-52.32 % DPPH inhibition		26	36			17
Peels hydrolysis	488 mg GAE / 100 g DW				-11.6	100		100	18
oil					IC_{50} - Acarbose 7.45 µg / mL extract 8.16 µg / mL	IC_{50} - Acarbose 8.44 µg / mL, extract 7.56 µg / mL		IC_{50} - 6.17 µg / mL	19
juices			24-5-42.0 % DPPH inhibition						12
young lemon shoots	8930 mg GAE / 100 g DW								20
juices	103 mg GAE / 100 mL	14 mg GAE / 100 mL	42.28 % DPPH inhibition	0.72 % ABTS inhibition					13
juices	222-236 mg GAE / 100 g	170-189 mg QE / 100 g	1558-1761 mM TE / 100 g						21
**Tomato**									
gordal variety *w*hole fruit	5632 mg GAE / 100 g DW	2152 mg QE / 100 g DW	8217 mM TE / 100 g DW	6389 mM TE / 100 g DW	34	52	63	97	This work
pulp homogenized in methanolic solution	48 mg GAE / 100 g								22
lyophilized samples	2080-3360 mg GAE / 100 g								23
locular gel and the serum	50-110 mg GAE / 100 g		210-380 mM TE / 100 g	350-700 mM TE / 100 g					24
QuEChERS	52 mg GAE / 100 g	24 mg QE / 100 g	98 mM TE / 100 g	114 mM TE / 100 g					25

lyophilized samples								IC50 - 8.5-57.4 mg / mL	26
Peels hydrolysis	365 mg GAE / 100 g				-37.7	42.5		100	18
juice	192 mg GAE / 100 g					72			27
lyophilized samples	784-1315 mg GAE / 100 g		IC50 - 146-175 μg / mL						28
freeze-dried frozen puree			664-1472 mM TE / 100 g	674-1650 mM TE / 100 g					29
freeze-dried frozen puree								30-40	30
**Uva-da-serra**									
Uva-da-serra whole fruit	24158 mg GAE / 100 g DW	13604 mg QE / 100 g DW	19150 mM TE / 100 g DW	85561 mM TE / 100 g DW	22	51	67	86	This work
myrtle oil								93.6	10
blueberry juice	128 mg GAE / 100 mL			63 mM TE / 100 mL	IC50 - 2.67 mg / mL	IC50 - 40.68 mg / mL			31
	7.5-24.5 mg GAE / 100 mL	0.2-12.6 mg QE / 100 mL							32
blueberry pulp homogenized in methanolic solution	185.6-929 mg GAE / 100 mL	39.5-64.6 mg QE / 100 mL							33
blueberry air dried, in ethanol,	1642-4442 mg GAE / 100 g DW	140-922 mg QE / 100 g DW	IC50 - 141-263 μg / mL			IC50 - Acarbose 31 μg / mL,			34

methanol and water extractions						extract 301-591 µg / mL		
lyophilized	885 mg GAE / 100 g DW	1467 mg RuE /100 g DW		180 mM TE / 100 g	IC_{50} - Acarbose 20 µg / mL, extract 2630 µg / mL	IC_{50} - Acarbose 2060 µg / mL, extract 1030 µg / mL	IC_{50} - 9360 µg / mL	35
dehydrated at 105 °C	345-426 mg GAE / 100 g FW		11-12 mM TE / 100 g FW					36
blueberry water-based extraction	80-140 mg GAE / 100 g FW		70-80 % DPPH inhibition		50-90	55-80		37

[1] % inhibition, unless indicated, [2] - mg GAE / 100 mL of juice.

References

1. da Costa, J. S.; da Cruz, E. d. N. S.; Setzer, W. N.; da Silva, J. K. d. R.; Maia, J. G. S.; Figueiredo, P. L. B., Essentials Oils from Brazilian Eugenia and Syzygium Species and Their Biological Activities. *Biomolecules* **2020,** *10* (8), 1155.
2. Santos, D. N.; de Souza, L. L.; de Oliveira, C. A. F.; Silva, E. R.; de Oliveira, A. L., Arginase inhibition, antibacterial and antioxidant activities of Pitanga seed (Eugenia uniflora L.) extracts from sustainable technologies of high pressure extraction. *Food bioscience* **2015,** *12*, 93-99.
3. Oliveira, A. L.; Destandau, E.; Fougère, L.; Lafosse, M., Isolation by pressurised fluid extraction (PFE) and identification using CPC and HPLC/ESI/MS of phenolic compounds from Brazilian cherry seeds (Eugenia uniflora L.). *Food Chem.* **2014,** *145*, 522-529.
4. Vinholes, J.; Vizzotto, M., Synergisms in alpha-glucosidase inhibition and antioxidant activity of camellia sinensis l. kuntze and eugenia uniflora l. ethanolic extracts. *Pharmacognosy Res.* **2017,** *9* (1), 101.
5. Vinholes, J.; Lemos, G.; Lia Barbieri, R.; Franzon, R.; Vizzotto, M., In vitro assessment of the antihyperglycemic and antioxidant properties of araçá, butiá and Pitanga. Food Biosci 19: 92–100. 2017.
6. Vinholes, J.; Reis, S. F.; Lemos, G.; Barbieri, R. L.; de Freitas, V.; Franzon, R. C.; Vizzotto, M., Effect of in vitro digestion on the functional properties of Psidium cattleianum Sabine (araçá), Butia odorata (Barb. Rodr.) Noblick (butiá) and Eugenia uniflora L.(pitanga) fruit extracts. *Food Funct.* **2018,** *9* (12), 6380-6390.
7. Siebert, D. A.; de Mello, F.; Alberton, M. D.; Vitali, L.; Micke, G. A., Determination of acetylcholinesterase and α-glucosidase inhibition by electrophoretically-mediated microanalysis and phenolic profile by HPLC-ESI-MS/MS of fruit juices from Brazilian Myrtaceae Plinia cauliflora (Mart.) Kausel and Eugenia uniflora L. *Nat. Prod. Res.* **2020,** *34* (18), 2683-2688.
8. Correia, R. T.; Borges, K. C.; Medeiros, M. F.; Genovese, M. I., Bioactive compounds and phenolic-linked functionality of powdered tropical fruit residues. *Food Sci. Technol. Int.* **2012,** *18* (6), 539-547.
9. Monteiro, J. R. B.; Ardisson, J. S.; Athaydes, B. R.; Gonçalves, R. d. C. R.; Rodrigues, R. P.; Kuster, R. M.; Kitagawa, R. R., Anti-Helicobacter pylori and Anti-inflammatory Properties of Eugenia uniflora L. *Brazilian Archives of Biology and Technology* **2019,** *62*.

10. Senthil Kumar, K.; Gokila Vani, M.; Wang, C.-S.; Chen, C.-C.; Chen, Y.-C.; Lu, L.-P.; Huang, C.-H.; Lai, C.-S.; Wang, S.-Y., Geranium and lemon essential oils and their active compounds downregulate angiotensin-converting enzyme 2 (ACE2), a SARS-CoV-2 spike receptor-binding domain, in epithelial cells. *Plants* **2020,** *9* (6), 770.
11. Raspo, M. A.; Vignola, M. B.; Andreatta, A. E.; Juliani, H. R., Antioxidant and antimicrobial activities of citrus essential oils from Argentina and the United States. *Food Bioscience* **2020,** *36*, 100651.
12. Goldenberg, L.; Yaniv, Y.; Porat, R.; Carmi, N., Mandarin fruit quality: a review. *J. Sci. Food Agric.* **2018,** *98* (1), 18-26.
13. Sicari, V.; Pellicanò, T. M.; Giuffrè, A. M.; Zappia, C.; Capocasale, M., Bioactive compounds and antioxidant activity of citrus juices produced from varieties cultivated in Calabria. *Journal of Food Measurement and Characterization* **2016,** *10* (4), 773-780.
14. Gironés-Vilaplana, A.; Moreno, D.; García-Viguera, C., Phytochemistry and biological activity of Spanish citrus fruits. Food Funct 5: 764–772. 2014.
15. Hussein, S.; Kaluf, A.; Ahmed, Y.; Ahmed, B.; Iyad, A., DETERMINATION OF INHIBITION ACTIVITY OF α-AMYLASE ENZYME, ANTIOXIDANT ACTIVITY, ANTIBACTERIAL Activity AND PHENOLIC COMPOUNDS BY USING SOME MEDICAL PLANTS. *The Iraqi Journal of Agricultural Science* **2020,** *51* (1), 411-421.
16. Tejpal, S.; Wemyss, A. M.; Bastie, C. C.; Klein-Seetharaman, J., Lemon Extract Reduces Angiotensin Converting Enzyme (ACE) Expression and Activity and Increases Insulin Sensitivity and Lipolysis in Mouse Adipocytes. *Nutrients* **2020,** *12* (8), 2348.
17. Lu, S.-Y.; Chu, Y.-L.; Sridhar, K.; Tsai, P.-J., Effect of ultrasound, high-pressure processing, and enzymatic hydrolysis on carbohydrate hydrolyzing enzymes and antioxidant activity of lemon (Citrus limon) flavedo. *LWT* **2021,** *138*, 110511.
18. Alu'datt, M. H.; Rababah, T.; Alhamad, M. N.; Al-Mahasneh, M. A.; Ereifej, K.; Al-Karaki, G.; Al-Duais, M.; Andrade, J. E.; Tranchant, C. C.; Kubow, S.; Ghozlan, K. A., Profiles of free and bound phenolics extracted from Citrus fruits and their roles in biological systems: content, and antioxidant, anti-diabetic and anti-hypertensive properties. *Food Funct.* **2017,** *8* (9), 3187-3197.
19. Oboh, G.; Olasehinde, T. A.; Ademosun, A. O., Inhibition of enzymes linked to type-2 diabetes and hypertension by essential oils from peels of orange and lemon. *Int. J. Food Sci. Prop.* **2017,** *20* (sup1), S586-S594.
20. Fabroni, S.; Ballistreri, G.; Amenta, M.; Romeo, F. V.; Rapisarda, P., Screening of the anthocyanin profile and in vitro pancreatic lipase inhibition by anthocyanin-containing extracts of fruits, vegetables, legumes and cereals. *J. Sci. Food Agric.* **2016,** *96* (14), 4713-4723.
21. Spínola, V.; Pinto, J.; Castilho, P. C., Identification and quantification of phenolic compounds of selected fruits from Madeira Island by HPLC-DAD–ESI-MSn and screening for their antioxidant activity. *Food Chem.* **2015,** *173*, 14-30.
22. Flores, I. R.; Vásquez-Murrieta, M. S.; Franco-Hernández, M. O.; Márquez-Herrera, C. E.; Ponce-Mendoza, A.; del Socorro López-Cortéz, M., Bioactive compounds in tomato (Solanum lycopersicum) variety saladette and their relationship with soil mineral content. *Food Chem.* **2021,** *344*, 128608.
23. Tan, S.; Ke, Z.; Chai, D.; Miao, Y.; Luo, K.; Li, W., Lycopene, polyphenols and antioxidant activities of three characteristic tomato cultivars subjected to two drying methods. *Food Chem.* **2021,** *338*, 128062.

24. Marcolongo, P.; Gamberucci, A.; Tamasi, G.; Pardini, A.; Bonechi, C.; Rossi, C.; Giunti, R.; Barone, V.; Borghini, A.; Fiorenzani, P., Chemical Characterisation and Antihypertensive Effects of Locular Gel and Serum of Lycopersicum esculentum L. var."Camone" Tomato in Spontaneously Hypertensive Rats. *Molecules* **2020,** *25* (16), 3758.
25. Aguiar, J.; Gonçalves, J. L.; Alves, V. L.; Câmara, J. S., Chemical Fingerprint of Free Polyphenols and Antioxidant Activity in Dietary Fruits and Vegetables Using a Non-Targeted Approach Based on QuEChERS Ultrasound-Assisted Extraction Combined with UHPLC-PDA. *Antioxidants* **2020,** *9* (4), 305.
26. Błaszczak, W.; Jeż, M.; Szwengiel, A., Polyphenols and inhibitory effects of crude and purified extracts from tomato varieties on the formation of advanced glycation end products and the activity of angiotensin-converting and acetylcholinesterase enzymes. *Food Chem.* **2020,** *314*, 126181.
27. Bot, F.; Anese, M.; Hungerford, G.; Lemos, M., Effect of high frequency ultrasounds on lycopene and total phenolic concentration, antioxidant properties and a-glucosidase inhibitory activity of tomato juice. *Italian Journal of Food Science* **2017,** *29* (3), 424-433.
28. Belović, M. M.; Gironés-Vilaplana, A.; Moreno, D. A.; Milovanović, I. L.; Novaković, A. R.; Karaman, M. A.; Ilić, N. M., Tomato (Solanum Lycopersicum L.) Processing Main Product (Juice) and By-Product (Pomace) Bioactivity Potential Measured as Antioxidant Activity and Angiotensin-Converting Enzyme Inhibition. *J. Food Process. Preserv.* **2016,** *40* (6), 1229-1237.
29. Sricharoen, P.; Techawongstein, S.; Chanthai, S., Determination of lipophilic and hydrophilic antioxidant activities in the crude extracts of ten varieties of tomatoes. *Asia-Pacific Journal of Science and Technology* **2015,** *20* (1), 66-74.
30. Biswas, D.; Uddin, M. M.; Dizdarevic, L. L.; Jørgensen, A.; Duttaroy, A. K., Inhibition of angiotensin-converting enzyme by aqueous extract of tomato. *Eur. J. Nutr.* **2014,** *53* (8), 1699-1706.
31. Zhong, H.; Zhao, M.; Tang, J.; Deng, L.; Feng, F., Probiotics-fermented blueberry juices as potential antidiabetic product: antioxidant, antimicrobial and antidiabetic potentials. *J. Sci. Food Agric.*
32. Yousefi, M.; Shadnoush, M.; Khorshidian, N.; Mortazavian, A. M., Insights to potential antihypertensive activity of berry fruits. *Phytother. Res.* **2021,** *35* (2), 846-863.
33. Tian, S.; Li, X.; Zang, S.; Jin, Y.; Zhang, Z.; Yu, Y., α-Amylase and tyrosinase inhibitory activities, phenolic contents, and antioxidant capacities of wild and cultivated blueberries. *J. Food Process. Preserv.* **2021,** *45* (1), e15087.
34. Barut, B.; Barut, E. N.; Engin, S.; Özel, A.; Sezen, F. S., Investigation of the Antioxidant, α-Glucosidase Inhibitory, Anti-inflammatory, and DNA Protective Properties of Vaccinium arctostaphylos L. *Turkish Journal of Pharmaceutical Sciences* **2019,** *16* (2), 175.
35. Spínola, V.; Pinto, J.; Castilho, P. C., Hypoglycemic, anti-glycation and antioxidant in vitro properties of two Vaccinium species from Macaronesia: A relation to their phenolic composition. *J. Funct. Foods* **2018,** *40*, 595-605.
36. Carvalho, M. J.; Gouveia, C. S.; Vieira, A. C.; Pereira, A. C.; Carvalho, M. Â.; Marques, J. C., Nutritional and phytochemical composition of Vaccinium padifolium Sm wild berries and radical scavenging activity. *J. Food Sci.* **2017,** *82* (11), 2554-2561.
37. Sarkar, D.; Agustinah, W.; Woods, F.; Coneva, E.; Vinson, E.; Shetty, K., In vitro screening and evaluation of phenolic antioxidant-linked anti-hyperglycemic functions of rabbit-eye blueberry (Vaccinium ashei) cultivars. *Journal of Berry Research* **2017,** *7* (3), 163-177.

Figure S1: Correlation dendograms obtained for the selected extracts.

A. Pitanga seeds

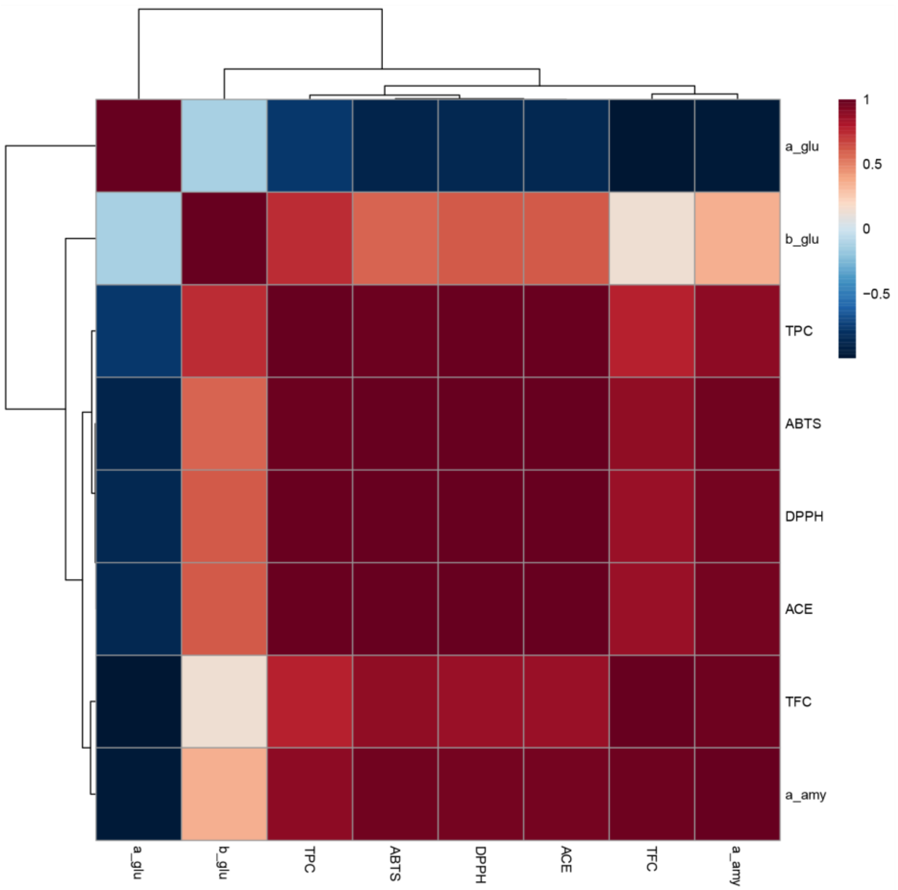

B. Pitanga pulp

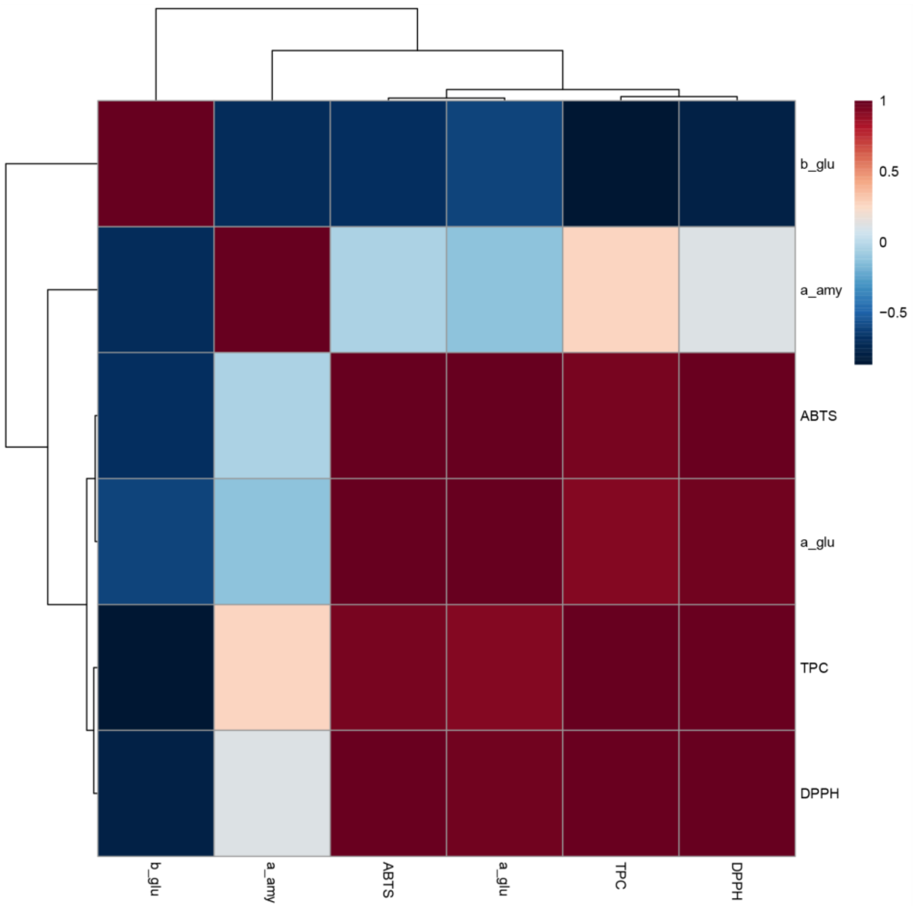

C. Lemon peel

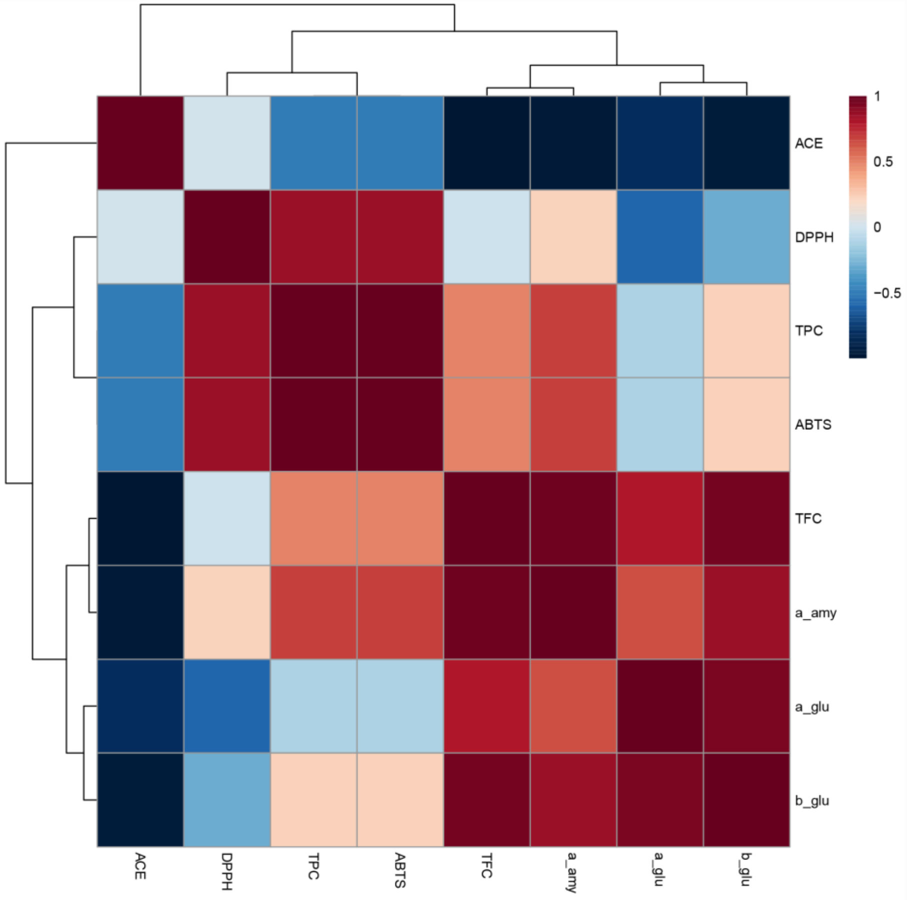

D. Lemon juice

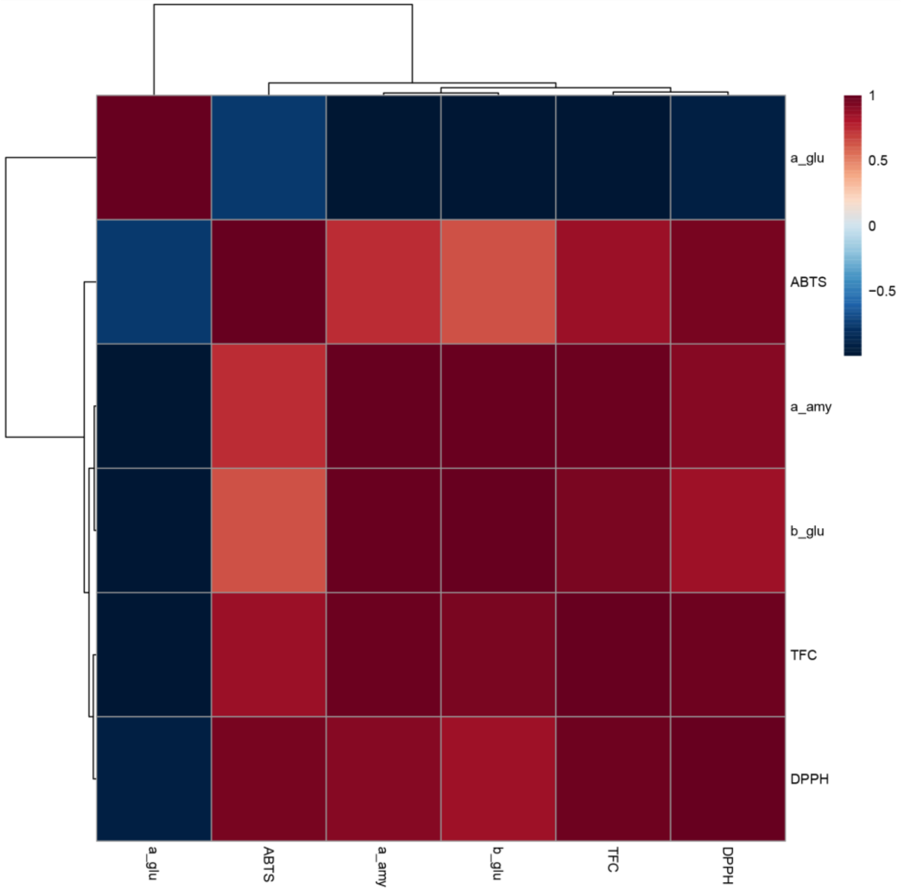

E. tangerine peel

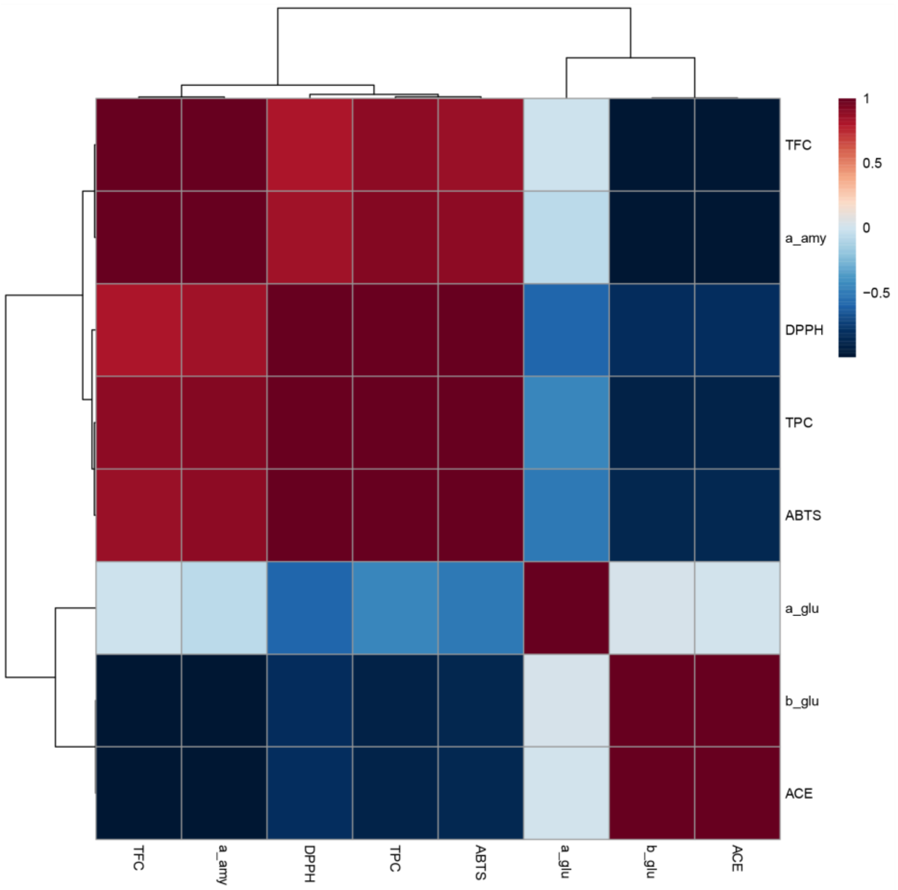

F. tangerine juice

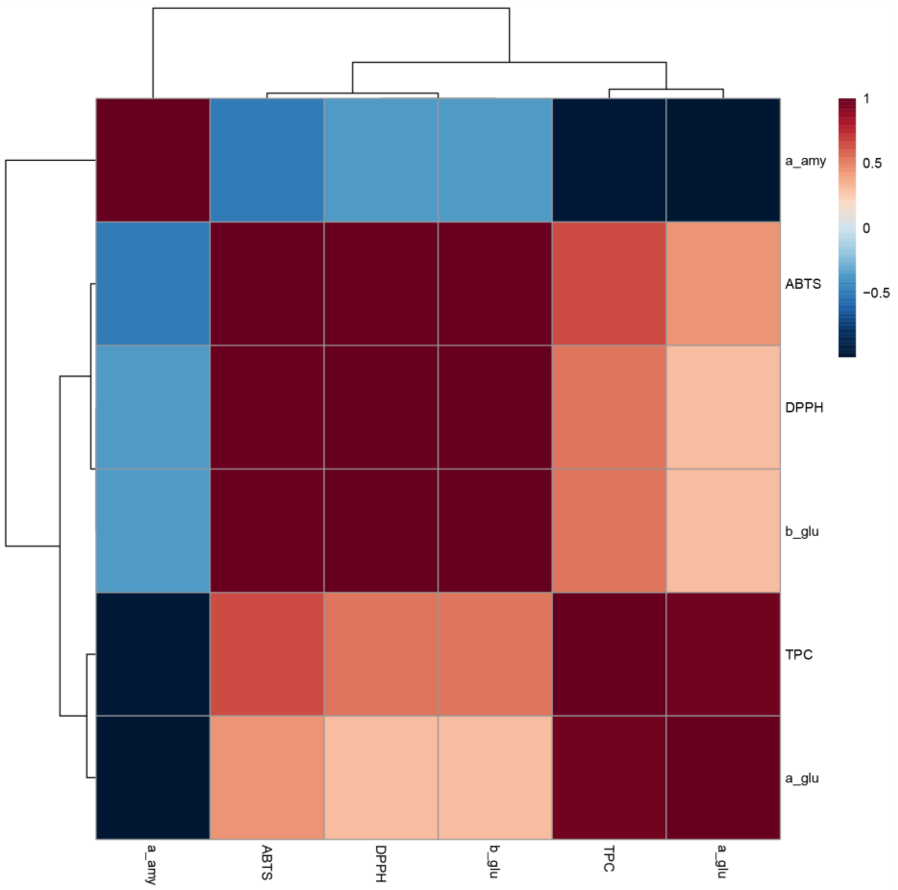

E. uva-da-serra

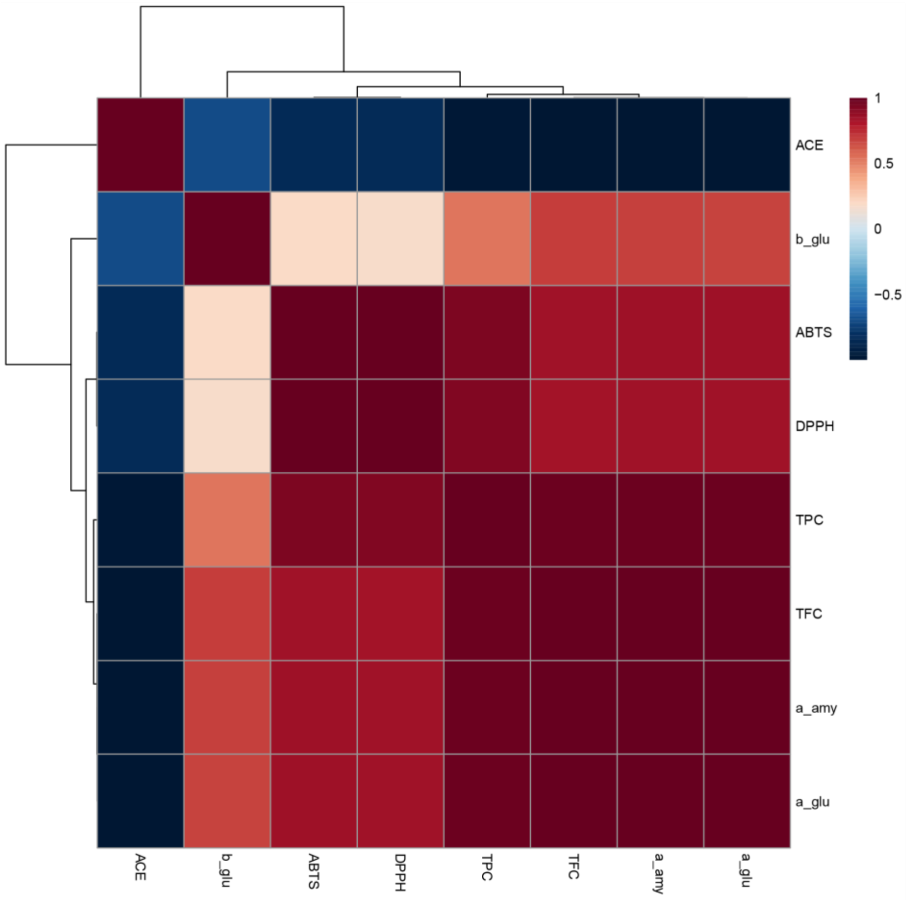

F. tomato

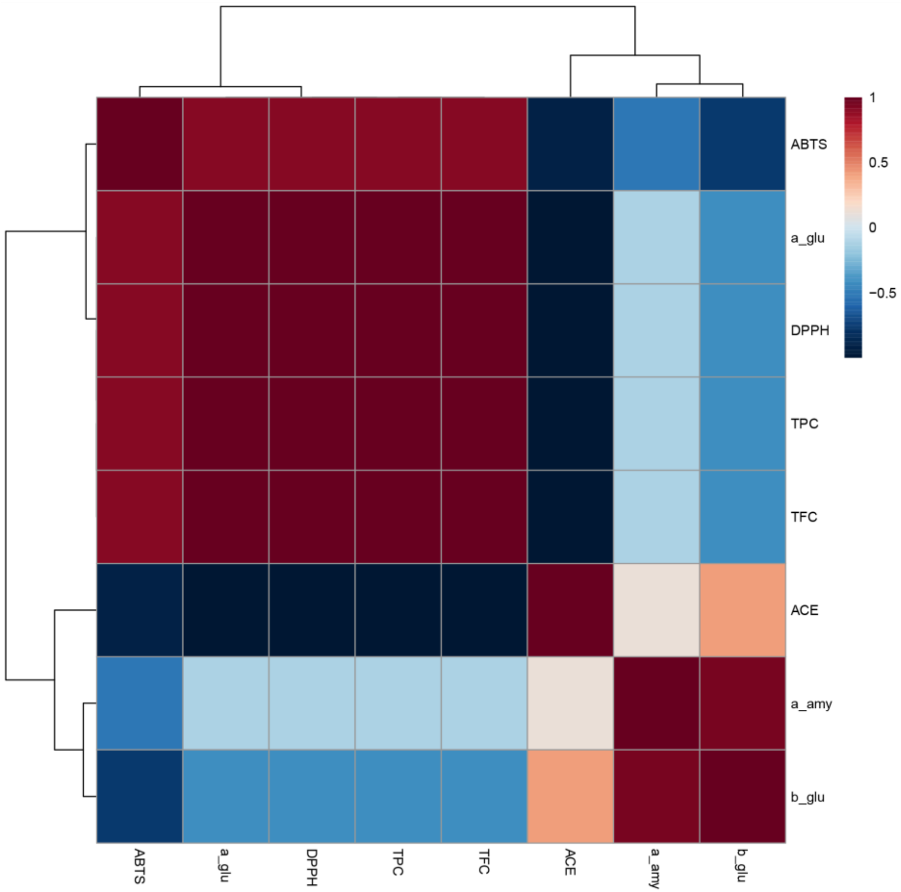

Supplement: Supplementary file 1 [file molecules-26-04202-s001.zip › molecules-1286302-supplementary.pdf]
